# Selective oestrogen receptor modulators and Alzheimer´s disease: a real-world pharmacovigilance study

**DOI:** 10.1007/s00228-026-04012-y

**Published:** 2026-02-18

**Authors:** María Teresa Yuste, Elena Badillo, Juan Sebastián Galecio, Pedro Marín

**Affiliations:** 1 Department of Pharmacology, University of Murcia, Campus de Espinardo, Murcia, 30100 Spain; 2 Escuela de Medicina Veterinaria. Colegio de Ciencias de la Salud, Universidad San Francisco de Quito, Cumbayá, EC 170157 Ecuador

**Keywords:** Pharmacovigilance, Selective oestrogen receptor modulators, Dementia, Alzheimer’s disease

## Abstract

**Purpose:**

Selective oestrogen receptor modulators (SERMs) are a standard treatment for breast cancer and osteoporosis. Alzheimer’s disease (AD) is a progressive neurodegenerative disorder that is a serious public health concern. This study aimed to identify potential pharmacovigilance signals related to dementia and AD for SERMs in menopausal and postmenopausal women.

**Methods:**

To investigate this possible association, a disproportionality analysis was performed in VigiBase, the World Health Organization’s (WHO) global database of individual case safety reports (ICSRs). Disproportionality was quantified using the reporting odds ratio (ROR).

**Results:**

We found risk of reporting dementia for tamoxifen [ROR = 1.74 (1.23–2.45)] and raloxifene [ROR = 1.63 (1.05–2.53)] and AD for raloxifene [ROR = 5.12 (3.26–8.05)]. No statistically significant association was detected for fulvestrant and bazedoxifene with dementia or AD. Most of the reports were severe and affected women over the age of 75. The latency of the reactions was predominantly long, suggesting that dementia is a late-onset reaction. The duration of treatment varied from a few months to more than three years, which may indicate that long-term exposure to SERMs is not necessary to develop dementia associated with these drugs.

**Conclusion:**

Signals of disproportionate reporting have been observed between the incidence of dementia and AD with SERMs. There are conflicting results from the different studies that have been conducted on the relationship between these drugs and AD. More research is needed to find out what factors determine the risk of cognitive impairment associated with SERMs.

## Introduction

Dementia is a major health problem given the ageing population, with Alzheimer’s disease (AD) being the most common among the elderly. This insidious onset neurodegenerative disease causes progressive deterioration of behavioural and cognitive functions such as memory, comprehension, language, attention, reasoning and judgement [1]. Two-thirds of clinically diagnosed cases of dementia and AD are women, with women’s greater longevity being the main reason for this gender difference, as the risk of developing dementia increases with age [2, 3].

Selective estrogen receptor modulators (SERMs) are currently used as a treatment for breast cancer, osteoporosis and postmenopausal symptoms, as these drugs have characteristics that allow them to act as estrogen agonists and antagonists, depending on the target tissue. After tamoxifen, others such as raloxifene, toremifene, fluvestrant, ospemifene and bazedoxifene have been developed and used for the treatment of these pathologies [4]. SERMs can be classified into different groups: (1) first generation SERMs such as tamoxifen and toremifene; (2) second generation SERMs such as raloxifene; (3) third generation SERMs such as ospemifene and bazedoxifene; and (4) steroid compounds such as fulvestrant [5].

The neurocognitive effects of endocrine therapy with selective oestrogen receptor modulators remain unclear. Previous studies suggest that declining oestrogen and progesterone levels after menopause may contribute to the development of cognitive impairment and dementia [6]. Estrogens exert neuroprotective effects, as demonstrated in several experiments with transgenic animal models and neuronal culture model systems [7]. They act as a neuroprotective agent by reducing Aβ and glutamate toxicity [8], enhancing synaptic plasticity, maintaining neurotrophic components, supporting transcription factor initiation, reducing brain inflammation and reducing tau protein hyperphosphorylation [9–12]. In addition, other studies have shown that women who undergo early surgical menopause have an increased risk of cognitive impairment and dementia [13].

Several studies link the use of SERMs with an increased risk of dementia [14, 15]. Other studies report a higher incidence of dementia in postmenopausal women with early breast cancer treated with tamoxifen than with aromatase inhibitors [16], highlighting that the latter have already shown signs of disproportionality for dementia, Alzheimer’s dementia and senile dementia in postmenopausal women [17]. However, recent experimental studies suggest that new SERMs may be promising treatments for AD due to their neuroprotective properties [18].

Databases for reporting adverse events play a crucial role in uncovering potential signals or new adverse drug reactions. Therefore, the aim of this study was to assess a possible association between major SERMs and reports of dementia or Alzheimer’s dementia using VigiBase, the World Health Organization’s (WHO) global database of individual case safety reports (ICSRs).

## Methods

### Study desing

To detect a possible signal between exposure to any SERMs and dementia or AD, a case/non-case study was conducted using VigiBase. This study design is suitable for detecting a potential signal between an adverse drug reaction and exposure to a specific drug by reporting the odds ratio (ROR) [19].

### Data description, access and preprocessing

VigiBase is maintained by the Uppsala Monitoring Centre (UMC), the WHO Collaborating Centre for International Drug Monitoring. The suspected adverse reactions (ADR) were submitted by reporters from the 150 member countries of the WHO International Drug Monitoring Programme (WHO PIDM) since 1968 [20]. Each ICSR contains, among other things, information on the patient, the medicinal product (classified as a suspect or a concomitant), the suspect ADR and the country that reported it [21]. Drugs are coded according to the Anatomical Therapeutic Chemical Classification (ATC) and ADRs are coded according to the Medical Dictionary for Regulatory Activities (MedDRA) [22].

Reports that were registered between the start of the programme on 1968 and 17 December 2024 were included in the analysis.

### Cases identification

The present study focused on ICSRs reported in women aged ≥ 45 years (menopausal and postmenopausal) from any country. ICSRs with unknown sex and age were excluded from the study. A deduplication process was applied before each analysis.

ICSRs for any SERM were reported as suspected, interacting or concomitant drugs were considered exposed. Fixed combinations were included. According the ATC classification, the following categories and active ingredients were selected: L02BA anti-oestrogens (L02BA01 Tamoxifen, L02BA02 Toremifene and L02BA03 Fulvestran) and G03XC selective oestrogen receptor modulators (G03XC01 Raloxifene, G03XC02 Bazedoxifene and G03XC05 Ospemifene).

The identification of the ADRs of interest was facilitated by the implementation of standardised MedDRA queries (SMQs) from MedDRA. Consequently, ICSRs containing a term from the SMQ ‘Dementia’ (MedDRA [version 27.1]) were designated as cases. The SMQ “Dementia” comprises the following Preferred Terms (PTs): “Clinical dementia rating scale score abnormal”, “Dementia”, “Dementia Alzheimer’s type”, “Dementia Alzheimer’s type, uncomplicated”, “Dementia Alzheimer’s type, with delirium”, “Dementia Alzheimer’s type, with delusions”, “Dementia Alzheimer’s type, with depressed mood”, “Dementia with Lewy bodies”, “Early onset familial Alzheimer’s disease”, “Frontotemporal dementia”, “Hippocampal atrophy”, “Mini mental status examination abnormal”, “Mixed dementia”, “Presenile dementia”, “Progressive supranuclear palsy”, “Senile dementia” and “Vascular dementia”. The ICSRs registered under the following terms have been excluded from the present study: “Corticobasal degeneration”, “Creutzfeldt-Jakob disease”, “Hippocampal sclerosis”, “Korsakoff’s syndrome”, “Prion disease”, “Scatolia” and “Variant Creutzfeldt-Jakob disease”. The rationale behind this exclusion is that they are not related to Alzheimer’s dementia. Non-cases constituted all other reports (ICSRs) in Vigibase during the same period, occurring in women aged over 45 years. VigiBase does not capture the diagnostic criteria used by reporters. Dementia-related Preferred Terms reflect the reporter’s clinical assessment and MedDRA coding, without information on whether standardized diagnostic tools were applied.

### Statistical methods

Descriptive statistics were calculated for the demographic (age group and origin of report) and clinical (seriousness, reaction outcome, time to onset, dechallenge outcome, rechallenge outcome and duration of drug treatment) characteristics of each report of interest using Microsoft Excel software.

A disproportionality analysis (or case-non-case analysis) was performed to examine the association between SERMs and dementia and AD. This disproportionality analysis is a comparison between the proportion of adverse effects reported in a particular group and the proportion of the same effects reported in the control group. These analyses estimate disproportionality in reporting using the reporting odds ratio (ROR) as an indicator of disproportionate reporting. The ROR is an appropriate measure of association and serves as an odds ratio in case-control studies. When the lower bound of the 95% confidence interval (CI) was greater than 1.00, it indicated that specific adverse effects were reported more frequently than expected [23] and this can be interpreted as a pharmacovigilance signal if at least three cases were reported in the period of interest.

The disproportionality analysis was performed for each drug individually (tamoxifen, toremifene, fulvestran, raloxifene, bazedoxifene and ospemifene) and for the class of drugs at ATC level 4 anti-oestrogens (including tamoxifen, toremifene, and fulvestran) and selective oestrogen receptor modulators (raloxifene, bazedoxifene, and ospemifene together). Toremifene and ospemifene did not have any cases and were therefore not included in the study.

## Results

**Fig. 1:**
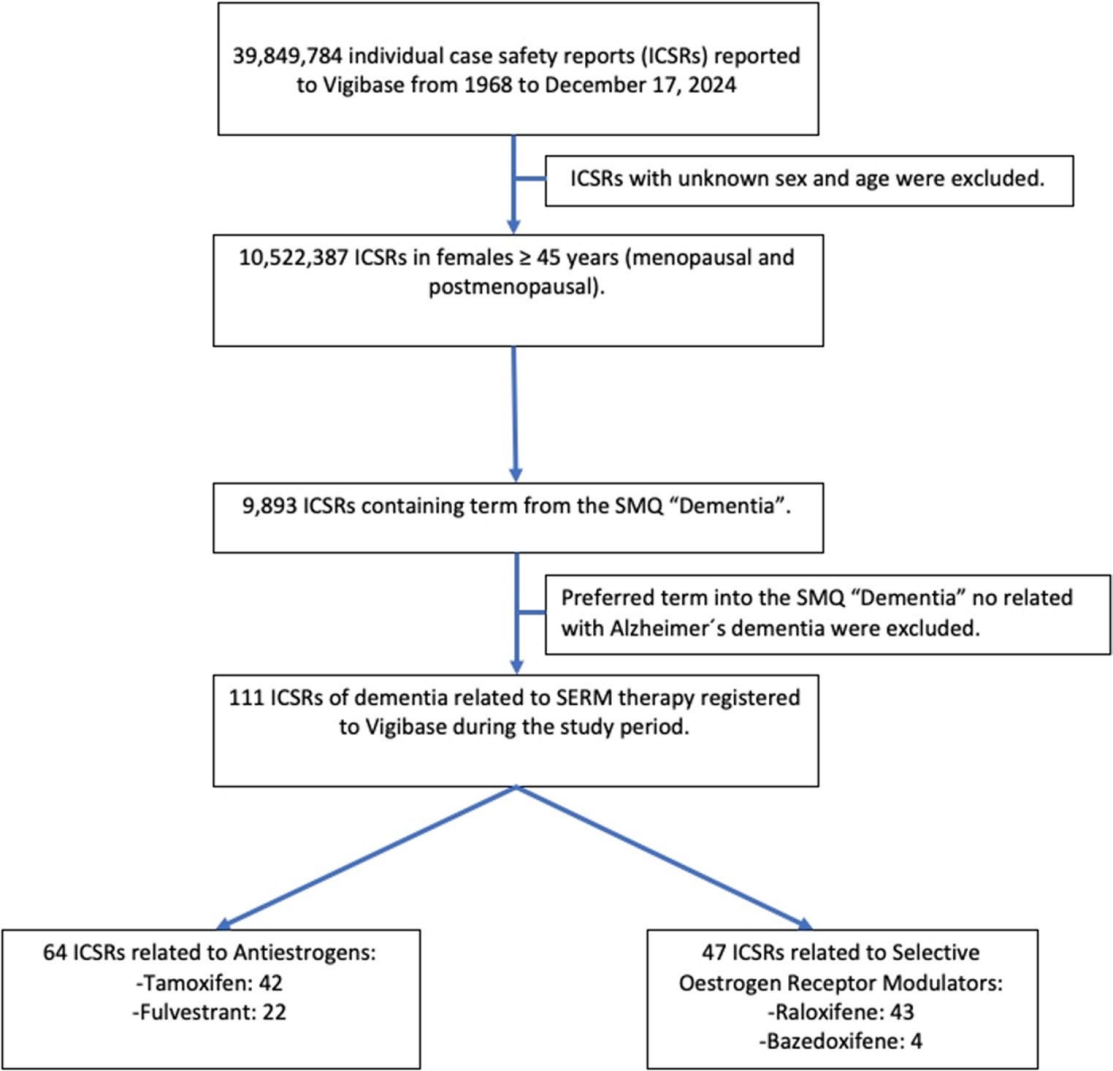
Flow chart of the pharmacovigilance study

Until 17 December 2024, of the total 39,849,784 reports in VigiBase, 10,522,387 were women aged ≥ 45 years, of which 111 cases were cases of SMQ dementia associated with SERMs. Of these ICSRs, 42 included tamoxifen, 22 fulvestrant, 43 raloxifene and 4 were related to bazedoxifene (Fig. 1).

The descriptive characteristics of the events related to antiestrogens are shown in Table 1. Compared with other age groups, patients aged ≥ 75 years were most likely to develop dementia after antiestrogen use (tamoxifen *n* = 18 (43%) and fulvestrant *n* = 11 (50%)). The majority of reports came from Americas and were serious for both drugs. Regarding the outcome of the reaction, in most cases where it was known, for the two drugs was not recovered. With tamoxifen, the latency periods were long in the known cases, more than a year. For fulvestrant, the latency period was only known in one case, 56 days. For tamoxifen, the effect of drug withdrawal in known cases was either no effect (*n* = 3, 7%) or decreased reaction (*n* = 2, 5%). In the case of fulvestrant, this effect was mostly unknown and in two cases no effect was observed. There are no data available on re-exposure to either of these drugs. Most known tamoxifen treatments were long (1–3 years: *n* = 2, 5%; ≥3 years *n* = 3, 7%). For fulvestrant, the duration of treatment was only known in two cases (≤ 1 year: *n* = 1, 5%; 1–3 years: *n* = 1, 5%). Tamoxifen was reported as the suspected drug in 22 of the 42 reports.

**Table 1:** Characteristics of the reported cases of dementia in women aged 45 and over who were taking antiestrogens: Tamoxifen and fulvestrant. (VigiBase, 1968 – December 2024)

	Tamoxifen	Fulvestrant
Total cases (suspected) – no.	42 (22)	22 (4)
Age group – no. (%)		
45–64 years	9 (21%)	4 (18%)
65–74 years	15 (36%)	7 (32%)
≥ 75 years	18 (43%)	11 (50%)
Origin of reports – no. (%)		
Americas	32 (76%)	17 (77%)
Asia	2 (5%)	3 (14%)
Europe	8 (19%)	2 (9%)
Oceania	0 (0%)	0 (0%)
Serious– no. (%)		
Yes	24 (57%)	21 (95%)
No	2 (5%)	1 (5%)
Unknown	16 (38%)	0 (0%)
Reaction outcome– no. (%)		
Fatal	4 (10%)	0 (0%)
Not recovered	8 (19%)	6 (27%)
Recovered/recovering	1 (2%)	1 (5%)
Recovered with sequelae	6 (14%)	0 (0%)
Died – unrelated to reaction	0 (0%)	0 (0%)
Unknown	23 (55%)	15 (68%)
Time to onset– no. (%)		
≤ 15 days	0 (0%)	0 (0%)
16–30 days	0 (0%)	0 (0%)
31–90 days	0 (0%)	1 (5%)
91–365 days	0 (0%)	0 (0%)
> 1 year	3 (7%)	0 (0%)
Unknown	39 (93%)	21 (95%)
Dechallenge outcome– no. (%)		
Fatal	0 (0%)	0 (0%)
No effect observed	3 (7%)	2 (9%)
Not applicable	16 (38%)	0 (0%)
Reaction abated	2 (5%)	0 (0%)
Unknown	21 (50%)	20 (91%)
Rechallenge outcome– no. (%)		
Not applicable	1 (2%)	0 (0%)
Unknown	41 (98%)	22 (100%)
Drug treatment duration– no. (%)		
≤ 1 year	3 (7%)	1 (5%)
1-3years	2 (5%)	1 (5%)
≥ 3 years	3 (7%)	0 (0%)
Unknown	34 (81%)	20 (90%)

Table 2 shows the characteristics of selective oestrogen receptor modulators notifications. For raloxifene, almost all ICSRs occurred in the Americas (*n* = 33, 77%). For bazedoxifene, there are only cases in Asia. The majority of cases reported were in patients aged ≥ 75 years and were serious for both drugs. In the known cases, the outcome of the reaction was mostly unrecoverable for either. In the reports on raloxifene, the latency period varied from one month to more than a year. Only one case was reported for bazedoxifene, and it was a long one, more than a year. Data on the effect of drug withdrawal are only available for reports involving raloxifene; in six cases no effect was observed and in four cases the response was attenuated. In none of the cases were there data on re-exposure. The duration of treatment varied from months to more than three years, according to the information provided.

**Table 2:** Characteristics of the reported cases of dementia in women aged 45 and over who were taking selective oestrogen receptor modulators: raloxifene and bazedoxifene. (VigiBase, 1968– December 2024)

	Raloxifene	Bazedoxifene
Total cases (suspected) – no.	43 (15)	4 (3)
Age group – no. (%)		
45–64 years	3 (7%)	0 (0%)
65–74 years	15 (35%)	0 (0%)
≥ 75 years	25 (58%)	4 (100%)
Origin of reports – no. (%)		
Americas	33 (77%)	0 (0%)
Asia	7 (16%)	4 (100%)
Europe	2 (5%)	0 (0%)
Oceania	1 (2%)	0 (0%)
Serious– no. (%)		
Yes	32 (74%)	3 (75%)
No	5 (12%)	1 (25%)
Unknown	6 (14%)	0 (0%)
Reaction outcome– no. (%)		
Fatal	0 (0%)	0 (0%)
Not recovered	9 (21%)	1 (25%)
Recovered/recovering	7 (16%)	0 (0%)
Recovered with sequelae	0 (0%)	0 (0%)
Died – unrelated to reaction	0 (0%)	0 (0%)
Unknown	27 (63%)	3 (75%)
Time to onset– no. (%)		
≤ 15 days	0 (0%)	0 (0%)
16–30 days	0 (0%)	0 (0%)
31–90 days	2 (5%)	0 (0%)
91–365 days	3 (7%)	0 (0%)
> 1 year	1 (2%)	1 (25%)
Unknown	37 (86%)	3 (75%)
Dechallenge outcome– no. (%)		
Fatal	0 (0%)	0 (0%)
No effect observed	6 (14%)	0 (0%)
Not applicable	1 (2%)	0 (0%)
Reaction abated	4 (9%)	0 (0%)
Unknown	32 (74%)	4 (100%)
Rechallenge outcome– no. (%)		
Not applicable	1 (2%)	0 (0%)
Unknown	42 (98%)	4 (100%)
Drug treatment duration– no. (%)		
≤ 1 year	5 (12%)	0 (0%)
1-3years	0 (0%)	0 (0%)
≥ 3 years	3 (7%)	1 (25%)
Unknown	35 (81%)	3 (75%)

A potential safety signal of disproportionality of dementia after antioestrogen treatment (50 cases; ROR 1.51;95%CI 1.15–2.00.15.00) was detected (Table 3). For the antiestrogen class, only for tamoxifen we found a statistically significant disproportionality with one of the preferred terms studied, dementia (33 cases; ROR 1.74;95% CI 1.23–2.45). For these associations, the information component (IC) and its 95% lower limit were > 0, also an indication of disproportionality.

**Table 3:** Reporting odds ratio (ROR) for the association between “Dementia” and antiestrogens, restricted to women ≥ 45 years (VigiBase, 1968 to December 2024)

Adverse Reaction (total cases)	Tamoxifen (*n* = 27,693)		Fulvestrant (*n* = 20,722)		All antiestrogens (*n* = 48,142)	
	*n*	ROR (CI 95%)	*n*	ROR (CI 95%)	*n*	ROR (CI 95%)
Dementia (7,236)	33	1.74 (1.23–2.45)	18	1.26 (0.80–2.00.80.00)	50	1.51 (1.15–2.00.15.00)
Dementia Alzheimer´s type (2,200)	8	1.38 (0.69–2.77)	4	0.92 (0.35–2.46)	11	1.09 (0.61–1.98)
Senile dementia (158)	0	NA †	0	NA	0	NA
Dementia with Lewy bodies (128)	0	NA	0	NA	1	2.79 (0.39–20.05)
Frontotemporal dementia (79)	1	4.86 (0.68–34.93)	0	NA	0	NA
Vascular dementia (208)	0	NA	0	NA	0	NA
Terms grouped (9,893)‡	**42**	**1.62** **(1.19–2.19)**	**22**	**1.13** **(0.74–1.72)**	**62**	**1.37** **(1.07–1.76)**

†NA: not applicable; ‡More than one of the terms of interest may be included in an ICSR

In the disproportionality analyses performed for selective oestrogen receptor modulators, including raloxifene and bazedoxifene, we found signals of disproportionality with dementia (23 cases; ROR 1.55;95% CI 1.03–2.33) and Alzheimer’s type dementia (20 cases; ROR 4.46;95% CI 2.87–6.92) (Table 4). There was no evidence of disproportionality between bazedoxifene and the PTs studied. However, our analysis detected a large signal disproportionality between raloxifene and Alzheimer’s type dementia (19 cases; ROR 5.12; 95% CI 3.26–8.05) and another signal with dementia (20 cases; ROR 1.63; 95% CI 1.05–2.53). For the associations of selective oestrogen receptor modulators and raloxifene with Alzheimer’s dementia, the Information Component (IC) and its 95% lower limit were > 0, also indicating disproportionality.

**Table 4:** Reporting odds ratio (ROR) for the association between “Dementia” and selective oestrogen receptor modulators restricted to women ≥ 45 years (VigiBase, 1968 to December 2024)

Adverse Reaction (total cases)	Raloxifene (*n* = 17,887)		Bazedoxifene (*n* = 2,265)		All selective oestrogen receptor modulators (*n* = 21,632)	
	*n*	ROR (CI 95%)	*n*	ROR (CI 95%)	*n*	ROR (CI 95%)
Dementia (7,236)	20	1.63 (1.05–2.53)	3	1.93 (0.62–5.98)	23	1.55 (1.03–2.33)
Dementia Alzheimer´s type (2,200)	19	5.12 (3.26–8.05)	1	2.11 (0.29–15.01)	20	4.46 (2.87–6.92)
Senile dementia (158)	1	3.74 (0.52–26.72)	0	NA	1	3.09 (0.43–22.09)
Dementia with Lewy bodies (128)	2	9.32 (2.31–37.69)	0	NA	2	7.71 (1.91–31.15)
Frontotemporal dementia (79)	0	NA †	0	NA	0	NA
Vascular dementia (208)	2	5.70 (1.42–22.96)	0	NA	2	4.71 (1.17–18.97)
Terms grouped (9,893)‡	**43**	**2.57** **(1.90–3.47)**	**4**	**1.88** **(0.71–5.02)**	**47**	**2.32** **(1.74–3.09)**

†NA: not applicable; ‡More than one of the terms of interest may be included in an ICSR

**Fig. 2:**
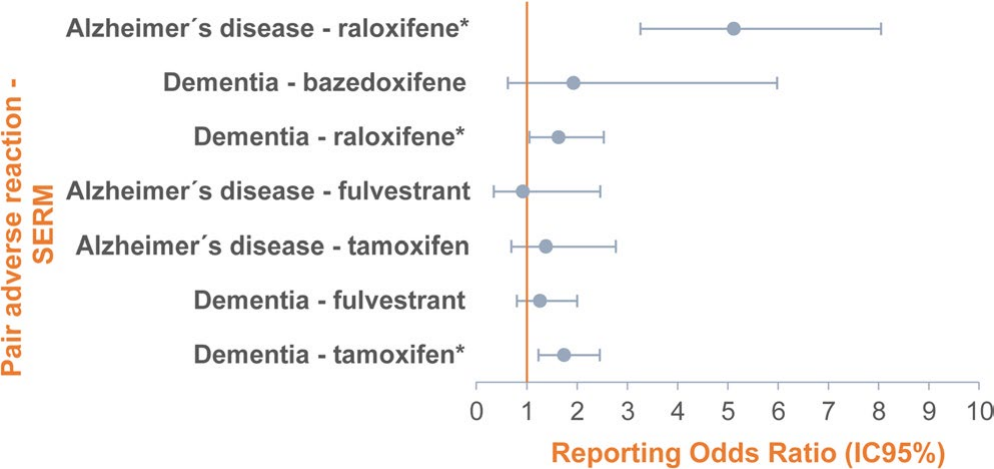
Disproportionality analysis illustrating the association between dementia and selective oestrogen receptor modulators (SERM). (* Signals of Disproportionate reporting)

Disproportionality analysis of cases of suspected SERM-induced dementia (Vigibase, 1968–2024) are presented in Fig. 2. An asterisk [*] denotes a potencial signal. Our analysis found no statistically significant association for fulvestrant and bazedoxifene with any of the selected dementia-related PTs.

## Discussion

### Key results

In the present study, the potential safety signals of dementia and AD following SERM treatment were investigated using VigiBase, the WHO global database of ICSRs containing more than 39 million reports.

Most of the reports were severe and affected women over the age of 75, highlighting age as a risk factor for the development of SERM-associated dementia. Most of the notifications came from the Americas, with the exception of bazedoxifene, all of which came from Asia. Although non-recovery was the most common outcome for all the drugs studied, tamoxifen had a significant number of cases that recovered from the reaction. This may indicate that the aetiopathogenesis of adverse reactions to tamoxifen may be different from that of the other SERMs studied. Despite there was variability in the latency of the reactions, they were predominantly long, suggesting that dementia is a late-onset reaction. Withdrawal of the drug had no effect in most patients, but it was significant that a decrease in response was observed in 4 of the reports related to raloxifene and 2 associated with tamoxifen, which is an important fact for the association with dementia. In addition, tamoxifen was reported as a suspected drug in 22 of the 42 reports collected, another factor to be considered in favour of causality. The duration of treatment varied widely, from a few months to more than three years. This could mean that long-term exposure to SERMs is not necessary for the development of dementia and AD.

Our analysis in VigiBase has detected signals of disproportionality between the use of antiestrogens (tamoxifen and fulvestrant) and dementia as well as between the selective oestrogen receptor modulators (raloxifene and bazedoxifene) with dementia and AD.

Tamoxifen and raloxifene were associated with dementia in disproportionality analyses conducted for each drug individually. We found a significantly increased risk of AD in women treated with raloxifene and for this agent, in four cases the response was decreased after discontinuing the drug. This strengthens the link between raloxifene and AD.

A recent pharmacovigilance study conducted in VigiBase found an association between endocrine therapies used in breast cancer, including SERMs, and neurocognitive impairment [24] but there are conflicting results from studies that have looked at the link between dementia and SERM. While a number of studies in humans have shown that endocrine therapy resulted in a decline in cognitive abilities [14, 25–27], there are also studies showing that this drugs not have significant cognitive effect [28–34] and even others suggesting that selective estrogen receptor modulators may be a promising therapeutic option for AD [18].

A cohort study in 2024 of more than 18,000 women treated with hormonal therapies, including tamoxifen and raloxifene, found that while these drugs may have a protective effect against AD in black women and among women under the age of 75, there was variability in the effect depending on several factors, highlighting the need for further research. No significant association between hormone therapy and AD was found in other patient populations [35]. That controversial findings on SERM-related cognitive function in humans may be associated with the population of women studied (due to age, race, or other factors), the doses, duration, and type of SERM administered, the type of cognitive function tests used, and menopausal status.

AD is the most common neurodegenerative disease and one of the most common neurological disorder, a health condition with profound economic and social ramifications [36, 37]. With the increasing use of SERM therapy to treat breast cancer and osteoporosis, it is imperative to understand the potential effects of these drugs on cognition.

### Limitations

The present study is inherently limited by the case/non-case study design [19, 22, 38, 39]. The primary limitation is the underreporting of the outcome, which is a common occurrence in pharmacovigilance studies. In this study, the low number of dementia reports relative to the underlying population suggests substantial underreporting of chronic neurological outcomes, which may lead to inaccurate and unstable estimates of disproportionate occurrence.

Vigibase only receives reports of suspected adverse reactions, this does not mean that they are actually adverse reactions, as a causal relationship has not been demonstrated [19]. A further limitation of studies conducted in pharmacovigilance databases is that they are unable to control for other risk factors that may act as confounders in the reactions under investigation.

With regard to the specific adverse reaction studied, dementia and AD, a main bias should be considered: indication bias. As SERMs are prescribed to postmenopausal women, there are other risk factors for dementia exist, such as age and naturally low estrogen levels. To minimize this bias, disproportionality analysis was restricted to women over 45 years, so that the comparators are in the same conditions and have the same risk factors.

Furthermore, it should be noted that the diagnostic approach to dementia cannot be verified, as VigiBase does not record whether diagnoses were based on formal criteria or clinical judgement.

Another limitation was that information on treatment duration and latency was often missing, limiting the ability to assess temporal plausibility. For example, in the case of fulvestrant, treatment duration was available in only 2 of 22 cases and latency in only 1 case.

Subgroup analyses by dementia subtype were based on very small numbers and should be interpreted with caution, as they may not provide reliable estimates. Therefore, it is also important to note that the disproportionality analysis should be regarded as exploratory in the context of signal detection. The ROR values should be considered as signals and not as real risk values. Pharmacovigilance database studies permit the identification of potential risks, which can then be evaluated in more comprehensive studies at a later stage.

## Conclusions

This real-world pharmacoepidemiological study in a large population found signals of disproportionality for tamoxifen and raloxifene with dementia and another important signal for raloxifene and AD. The study revealed no statistically significant link between fulvestrant or bazedoxifene and the development of dementia or Alzheimer’s disease. These reactions usually affect women over the age of 75. Most cases had long latency periods and were considered serious adverse reactions. The duration of treatment does not seem to influence the development of the reaction, as this varied from case to case. These exploratory findings must be interpreted with caution due to underreporting, lacking clinical information and small sample sizes. Further research is required to confirm these results.

## Data Availability

The data that support the findings of this study are available from the Uppsala Monitoring Centre. The data supporting the conclusions of this article will be made available by the corresponding author upon reasonable request.

## Code availability

N/A.

## Declarations

### Statements

Vigibase is managed by the Uppsala Monitoring Centre (UMC), the World Health Organization (WHO) Collaborating Centre for International Drug Monitoring. The information comes from a variety of sources, and the probability that the suspected adverse effect is drug-related is not the same in all cases. The discussion and conclusions of this study are authors’ considerations and do not represent the opinion of the UMC or the WHO.
